# Supplementary figures and images for: Genome-Wide Identification and Characterization of the Soybean DEAD-Box Gene Family and Expression Response to Rhizobia
Source: Int J Mol Sci. 2022 Jan 20;23(3):1120. doi: 10.3390/ijms23031120 (PMC8835661; doi:10.3390/ijms23031120)

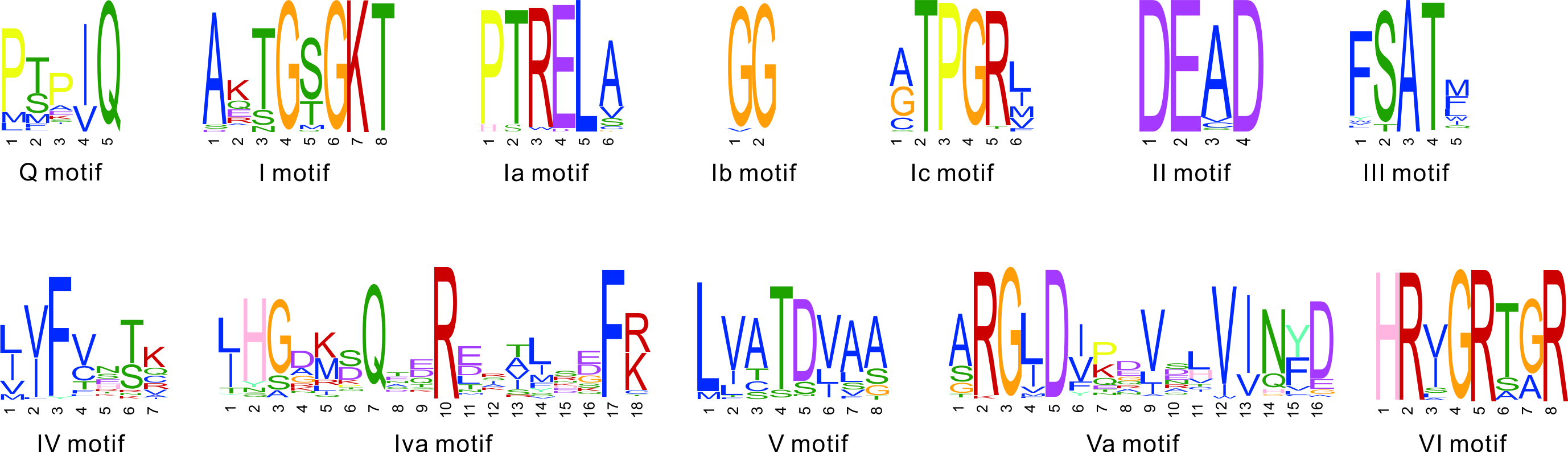

Supplement: Supplementary file 1 [file ijms-23-01120-s001.zip › FigureS1.tif]

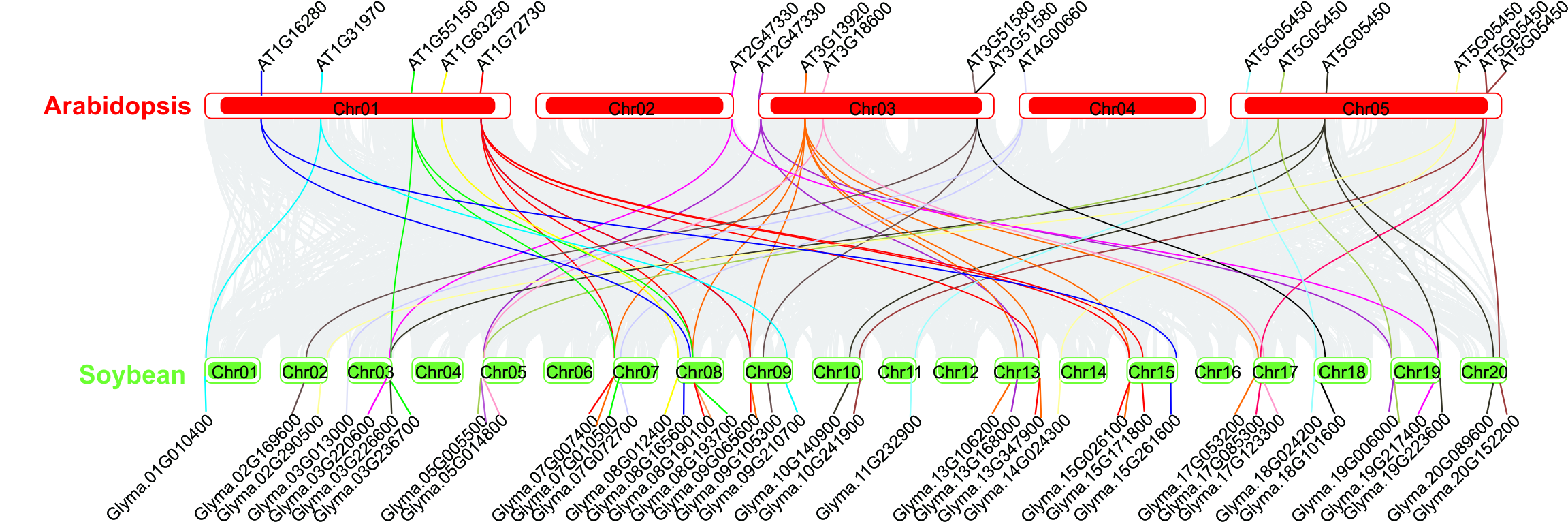

Supplement: Supplementary file 1 [file ijms-23-01120-s001.zip › FigureS2.tif]

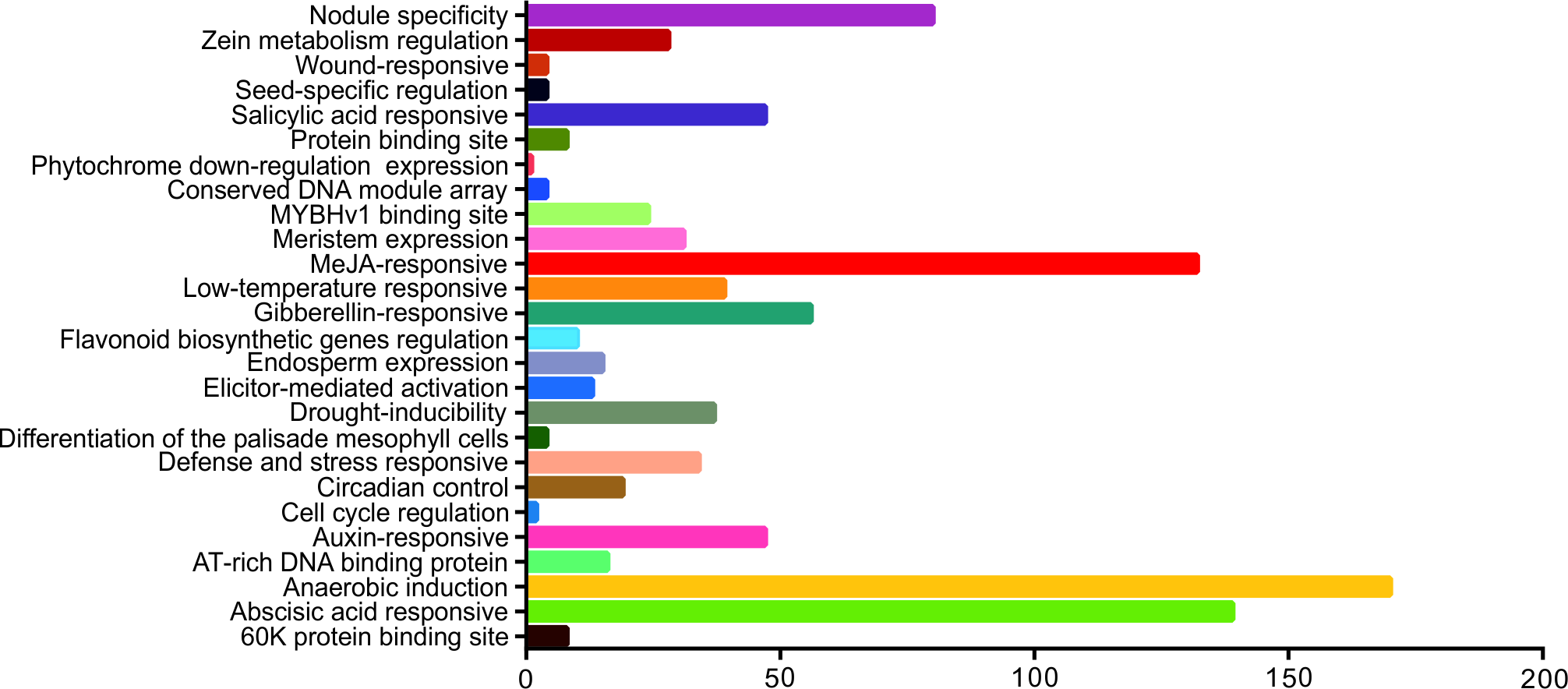

Supplement: Supplementary file 1 [file ijms-23-01120-s001.zip › FigureS3.tif]

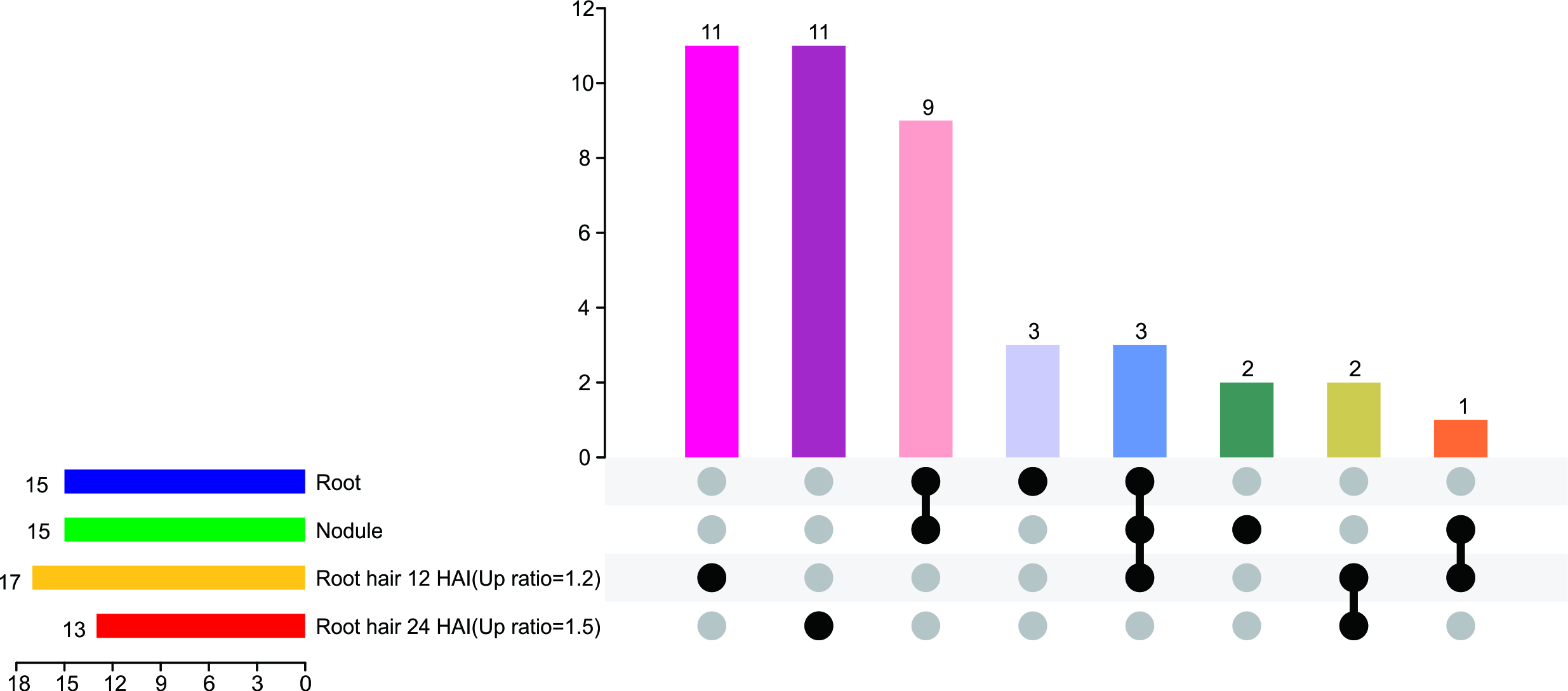

Supplement: Supplementary file 1 [file ijms-23-01120-s001.zip › FigureS4.tif]
